# Assessment of Simulated SARS-CoV-2 Infection and Mortality Risk Associated With Radiation Therapy Among Patients in 8 Randomized Clinical Trials

**DOI:** 10.1001/jamanetworkopen.2021.3304

**Published:** 2021-03-29

**Authors:** Shervin Tabrizi, Lorenzo Trippa, Daniel Cagney, Ayal A. Aizer, Shyam Tanguturi, Steffen Ventz, Geoffrey Fell, Jennifer R. Bellon, Harvey Mamon, Paul L. Nguyen, Anthony V. D’Amico, Daphne Haas-Kogan, Brian M. Alexander, Rifaquat Rahman

**Affiliations:** 1 Harvard Radiation Oncology Program, Boston, Massachusetts; 2 Department of Radiation Oncology, Dana-Farber/Brigham and Women’s Cancer Center, Harvard Medical School, Boston, Massachusetts; 3 Dana-Farber Cancer Institute, Department of Biostatistics and Computational Biology, Harvard School of Public Health, Boston, Massachusetts

## Abstract

**Importance:**

During the COVID-19 pandemic, cancer therapy may put patients at risk of SARS-CoV-2 infection and mortality. The impacts of proposed alternatives on reducing infection risk are unknown.

**Objective:**

To investigate how the COVID-19 pandemic is associated with the risks and benefits of standard radiation therapy (RT).

**Design, Setting, and Participants:**

This comparative effectiveness study used estimated individual patient–level data extracted from published Kaplan-Meier survival figures from 8 randomized clinical trials across oncology from 1993 to 2014 that evaluated the inclusion of RT or compared different RT fractionation regimens. Included trials were Dutch TME and TROG 01.04 examining rectal cancer; CALGB 9343, OCOG hypofractionation trial, FAST-Forward, and NSABP B-39 examining early stage breast cancer, and CHHiP and HYPO-RT-PC examining prostate cancer. Risk of SARS-CoV-2 infection and mortality associated with receipt of RT in the treatment arms were simulated and trials were reanalyzed. Data were analyzed between April 1, 2020, and June 30, 2020.

**Exposures:**

COVID-19 risk associated with treatment was simulated across different pandemic scenarios, varying infection risk per fractions (IRFs) and case fatality rates (CFRs).

**Main Outcomes and Measures:**

Overall survival was evaluated using Cox proportional hazards modeling under different pandemic scenarios.

**Results:**

Estimated IPLD from a total of 14 170 patients were included in the simulations. In scenarios with low COVID-19-associated risks (IRF, 0.5%; CFR, 5%), fractionation was not significantly associated with outcomes. In locally advanced rectal cancer, short-course RT was associated with better outcomes than long-course chemoradiation (TROG 01.04) and was associated with similar outcomes as RT omission (Dutch TME) in most settings (eg, TROG 01.04 median HR, 0.66 [95% CI, 0.46-0.96]; Dutch TME median HR, 0.91 [95% CI, 0.80-1.03] in a scenario with IRF 5% and CFR 20%). Moderate hypofractionation in early stage breast cancer (OCOG hypofractionation trial) and prostate cancer (CHHiP) was not associated with survival benefits in the setting of COVID-19 (eg, OCOG hypofractionation trial median HR, 0.89 [95% CI, 0.74-1.06]; CHHiP median HR, 0.87 [95% CI, 0.75-1.01] under high-risk scenario with IRF 10% and CFR 30%). More aggressive hypofractionation (FAST-Forward, HYPO-RT-PC) and accelerated partial breast irradiation (NSABP B-39) were associated with improved survival in higher risk scenarios (eg, FAST-Forward median HR, 0.58 [95% CI, 0.49-0.68]; HYPO-RT-PC median HR, 0.60 [95% CI, 0.48-0.75] under scenario with IRF 10% and CFR 30%).

**Conclusions and Relevance:**

In this comparative effectiveness study of data from 8 clinical trials of patients receiving radiation therapy to simulate COVID-19 risk and mortality rates, treatment modification was not associated with altered risk from COVID-19 in lower-risk scenarios and was only associated with decreased mortality in very high COVID-19–risk scenarios. This model, which can be adapted to dynamic changes in COVID-19 risk, provides a flexible, quantitative approach to assess the potential impact of treatment modifications and supports the continued delivery of standard evidence-based care with appropriate precautions against COVID-19.

## Key Points

**Question:**

How is the COVID-19 pandemic associated with risks and benefits of standard cancer therapy?

**Findings:**

In this comparative effectiveness study reanalyzing data from 8 randomized trials across oncology, radiation fractionation was not associated with outcomes in low COVID-19–risk scenarios. In higher-risk scenarios, aggressive hypofractionation was associated with a survival benefit, whereas moderate hypofractionation was not.

**Meaning:**

These findings suggest that the expected benefit of altering radiation therapy during the COVID-19 pandemic is associated with local pandemic factors and the specifics of treatment modification, and can be estimated using a quantitative framework based on completed randomized trials to support treatment decision-making.

## Teaser

This comparative effectiveness study investigates how the COVID-19 pandemic is associated with the benefits and risks of standard radiation therapy in simulated patients.

## Introduction

Cancer care remains essential during the COVID-19 pandemic but requires repeated contact with the health care system. People with cancer are at particularly high risk for COVID-19.^1,2,3^ Each interaction with the health care system during cancer therapy places patients at risk of SARS-CoV-2 infection and sequelae that could negatively affect survival. To mitigate these risks, multiple groups have published treatment guidelines advocating shorter treatment courses or omission of treatments with risk of immunosuppression.^4,5^ Randomized clinical trials (RCTs) that evaluated these treatment regimens were performed in non-pandemic settings. The extent to which these alternative courses should be reconsidered in a pandemic setting is unclear. There is an urgent need to integrate COVID-19–associated risks with existing evidence to understand the tradeoff of risks associated with cancer therapy in the COVID-19 era.

In this comparative effectiveness study, we developed a simulation-based model to reanalyze published RCTs with incorporation of the risk of COVID-19 infection associated with receipt of radiation therapy.^6^ This quantitative framework could help guide treatment decision-making and allow for characterization of infection risk associated with treatment. We applied this framework to several trials that have helped define the current standard of care in rectal, breast, and prostate cancer.

## Methods

This comparative effectiveness study used publicly available data from publications by using image analysis to reconstruct and estimate survival data from published figures. An estimated dataset of patient survival was generated based on a mathematical algorithm and did not use patient clinical trial data requiring informed consent. Given the methods, the project was determined to be exempt from review and informed consent by the Mass General Brigham institutional review board, as it did not meet criteria for human participant research. This study is reported in accordance with recommendations by the International Society for Pharmacoeconomics and Outcomes Research (ISPOR) reporting guideline.

### Study Inclusion and Data Acquisition

We selected a sample of 8 trials in rectal, breast, and prostate cancer, 3 common diseases sites with published, high-quality RCTs of different radiation fractionation approaches. To be included, publications were required to contain Kaplan-Meier curves for overall survival (OS) with at-risk tables and to report total numbers of death events. We included the Dutch TME trial (1996-1999)^7^ and the Trans-Tasman Radiation Oncology Group (TROG) 01.04 trial (2001-2006) in rectal cancer^8^; the Cancer and Leukemia Group B (CALGB) 9343 trial (1994-1999),^9^ Ontario Clinical Oncology Group (OCOG) hypofractionation trial (NCT00156052) (1993-1996),^10^ UK FAST-Forward trial (ISRCTN19906132) (2011-2014),^11^ and the National Surgical Adjuvant Breast and Bowel Project (NSABP) B-39 (NCT00103181) trial (2005-2013)^12^ in early stage breast cancer; and the Conventional or Hypofractionated High Dose Intensity Modulated Radiotherapy in Prostate Cancer (CHHiP) (CRUK/06/016, ISRCTN97182923) trial (2002-2011)^13^ and the Ultra-Hypofractionated vs Conventionally Fractionated Radiotherapy for Prostate Cancer (HYPO-RT-PC) (ISRCTN45905321) trial (2005-2015)^14^ in localized prostate cancer. Estimated individual patient–level data were extracted from published Kaplan-Meier survival curves as previously described.^15,16,17^ Outcome measures (hazard ratios [HRs] and OS estimates) and Kaplan-Meier figures from reconstructed data sets were compared with original publications for quality assurance (eFigure 1 in the Supplement).

### COVID-19 Mortality Incorporation

We simulated risk of SARS-CoV-2 infection using a binomial transmission model based on prior work.^6,18^ Two parameters were used to simulate mortality due to COVID-19: (1) the risk of SARS-CoV-2 infection per fraction of radiation received (ie, infection risk per fraction [IRF]) and (2) the risk of death of COVID-19 for patients who are infected (ie, case fatality rate [CFR]). IRF was assumed to be a constant, per-fraction risk, independent of prior fractions. Our model did not incorporate other possible infection risks (eg, other treatment modalities, community-acquired infection) except for risk per radiation fraction.

For each patient, we simulated the radiation fraction at which that patient was infected with a geometric random variable, with IRF representing the failure probability for each fraction. Patients for whom this value was less than the total number of fractions they received were considered to have been infected by SARS-CoV-2 during treatment (*n*). For the accelerated partial breast irradiation (APBI) arm in NSABP B-39, we conservatively simulated 10 fractions.^12^ We then simulated the number of deaths (*N_d_*) caused by COVID-19 using a binomial distribution with parameters *n* and death rate *P* = CFR, and selected *N_d_* individuals randomly from the pool of patients who were infected. These *N_d_* individuals had their survival time adjusted as follows: we assumed an approximately 4-week period between diagnosis and start of radiation and added the number of fractions on treatment until infection (based on the aforementioned simulations). We added a 5-day incubation period, and simulated time from symptom onset to death as a γ random variable with mean 18 days, based on published estimates.^19,20^ The risk of COVID-19–related death (simulated components) and the risk of non–COVID-19–related death (actual data) were distinct competing risks, and the occurrence of 1 of these 2 events became the censoring time point of the other.

### Parameter Values

In the general population, available estimates suggest an infection mortality rate of 4.3% for patients aged 70 to 79 years, 1.9% for patients aged 60 to 69 years, and 0.6% for patients aged 50 to 59 years, which are the age groups of patients with cancer who are enrolled in many RCTs.^19^ For illustrative pandemic scenarios, we drew on the current literature of COVID-19 in patients with cancer, which suggests that patients with cancer have a higher risk of death from COVID-19 compared with individuals without cancer.^21^ Current data suggest an approximately 2.5- to 5-fold higher rate of severe events and death in patients with cancer and reported CFRs of 13% to 28%.^22,23,24,25^ These reports, along with concern of higher mortality rates with active cancer therapy,^21^ informed our decision to simulate scenarios with 5%, 20%, and 30% COVID-19 CFRs. For daily risk with each fraction of radiotherapy, we simulated scenarios with 0.5% to 10% daily IRF. Given the many scenarios simulated, we focused on 3 risk scenarios for illustrative purposes: scenario A, with 0.5% IRF and 5% CFR; scenario B, with 5% IRF and 20% CFR; and scenario C, with 10% IRF and 30% CFR.

We expected scenario A to be representative of many places that are beyond an initial surge of infections and with persistent risk that is kept low with rigorous measures to minimize risks for patients and staff. Given the dynamic nature of pandemic spread, there are likely areas of accelerating outbreaks between scenario A and scenario B, a higher risk scenario. Scenario C is representative of an extreme, rare situation in which the health care system is overwhelmed and the infection risk is very high. Therefore, we consider scenario A as a lower-risk, most common pandemic setting, and scenarios B and C as higher-risk pandemic settings.

### Statistical Analysis

We generated 25 000 simulation replicates, adding COVID-19 risk to individual outcomes from RCTs, for each combination of daily risk of IRF and COVID-19 CFR. For each simulation, after adding COVID-19–related deaths to each trial arm, we performed Cox proportional hazards analysis for OS to obtain estimates of the HRs and 95% CIs. We reported the median HR and median values of the point estimates of the upper and lower 95% CIs, and median 5-year OS estimates across 25 000 bootstrap replicates for each scenario. In other words, we approximate study-specific point estimates and 95% CIs had the study occurred at a time with COVID-19–related risks. Analyses were conducted using R statistical software version 3.6.3 (R Project for Statistical Computing). Data were analyzed from April 1, 2020, to June 30, 2020.

## Results

Estimated individual patient-level data from 8 selected randomized clinical trials comprising 14 170 patients with breast, prostate or rectal cancer were analyzed.

### Preoperative Hypofractionated Radiation in Rectal Cancer

**Figure 1.**
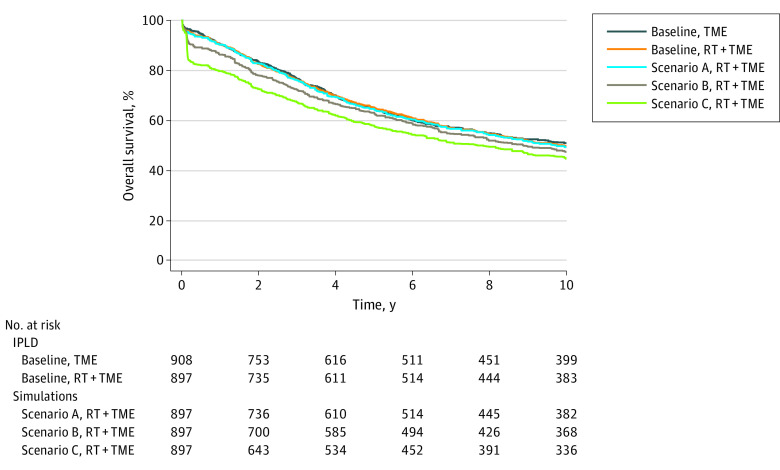
Overall Survival Outcomes From Simulations of the Dutch TME Trial^7^ Kaplan-Meier estimates of OS with total mesorectal excision (TME) vs TME and preoperative radiation therapy (TME + RT) at baseline (without COVID-19 risk) and under 3 scenarios (A, B, C) with increasing COVID-19 infection risk and case-fatality rate. Kaplan-Meier curves and number at risk values for scenarios A, B, and C are medians from 25 000 simulations. Scenario A indicates 0.5% infection risk per fraction and 5% case fatality rate; scenario B, 5% infection risk per fraction and 20% case fatality rate; scenario C, 10% infection risk per fraction and 30% case fatality rate; and IPLD, individual patient–level data.

The Dutch TME trial^7^ compared preoperative RT of 25 Gy in 5 fractions followed by total mesorectal excision (RT+TME) vs TME alone, and demonstrated a reduction in local recurrence rates of more than 50% with RT, without a difference in OS. In our reconstructed data set, prior to simulation of COVID-19 deaths, we obtained an HR of 0.99 (95% CI, 0.87-1.12) for OS, very close to the published trial. We reanalyzed the Dutch TME trial with simulation of COVID-19 risk under different pandemic scenarios (Figure 1; Table). OS remained similar between the 2 arms in lower-risk scenarios, with 5-year OS of 64% (95% CI, 61%-67%) in scenario A and 61% (95% CI, 58%-64%) in scenario B in the RT with total mesorectal excision (TME) arm. As pandemic risk increased, COVID-19–associated mortality was increasingly detrimental to OS, with a marked decrease in the highest-risk scenario (scenario C) to 56% (95% CI, 53%-59%) 5-year OS for RT with TME and a median HR of 0.80 (median 95% CI, 0.70-0.90) in favor of TME alone. More detailed evaluation across a range of parameter values demonstrated trends in expected HR for OS across a wide range of pandemic scenarios (eFigure 2 in the Supplement), with the most noticeable divergence in the highest risk scenarios (ie, IRF >5%; CFR >20%).

**Table.** Survival Analysis in Reanalyzed Randomized Trials With Incorporation of COVID-19 Associated Risks

Study	No.	HR (95% CI)				5-y overall survival, % (95% CI)			
		Baseline^a^	Scenario A^b^	Scenario B^c^	Scenario C^d^	Baseline^a^	Scenario A^b^	Scenario B^c^	Scenario C^d^
**Rectal cancer**									
Dutch TME: Van Gijn et al^7^ 2011 clinically resectable primary; cM0									
RT + TME	897	1 [Reference]	1 [Reference]	1 [Reference]	1 [Reference]	64 (61-67)	64 (61-67)	61 (58-64)	56 (53-59)
TME	908	0.99 (0.87-1.12)	0.99 (0.87-1.12)	0.91 (0.81-1.03)	0.80 (0.70-0.90)	63 (60-67)	63 (60-67)	63 (60-67)	63 (60-67)
TROG 01.04: Ngan et al,^8^ 2012 T3, N0-2, M0									
Chemo-RT (50.4 Gy/28 fx +5-fluorouracil)	161	1 [Reference]	1 [Reference]	1 [Reference]	1 [Reference]	70 (63-77)	69 (62-77)	59 (52-67)	50 (43-58)
RT (25 Gy/5 fx)	162	0.88 (0.59-1.31)	0.86 (0.58-1.28)	0.67 (0.47-0.97)	0.63 (0.45-0.88)	74 (67-82)	74 (67-82)	71 (64-79)	65 (58-73)
**Early stage breast cancer**									
CALGB 9343 Hughes et al,^9^ 2013; T1, N0, M0; ER+; age >70 y									
TamRT	317	1 [Reference]	1 [Reference]	1 [Reference]	1 [Reference]	85 (81-89)	85 (81-89)	72 (67-77)	61 (55-66)
Tam	319	1.06 (0.86-1.31)	1.05 (0.85-1.29)	0.79 (0.64-0.97)	0.62 (0.51-0.76)	85 (81-89)	85 (81-89)	85 (81-89)	85 (81-89)
OCOG hypofractionation: Whelan et al,^10^ 2010; T1-2, N0, M0									
RT (50 Gy/25 fx)	612	1 [Reference]	1 [Reference]	1 [Reference]	1 [Reference]	94 (92-96)	93 (91-95)	80 (77-83)	67 (64-71)
RT (42.5 Gy/16 fx)	622	0.93 (0.71-1.21)	0.92 (0.71-1.20)	0.85 (0.69-1.05)	0.89 (0.75-1.06)	94 (92-96)	93 (92-95)	83 (80-86)	71 (67-75)
FAST-Forward: Murray Brunt et al,^11^ 2020; T1-3, N0-1, M0									
RT (40 Gy/15 fx)	1361	1 [Reference]	1 [Reference]	1 [Reference]	1 [Reference]	94 (93-96)	94 (93-95)	84 (82-86)	72 (70-74)
RT (26 Gy/5 fx)	1368	0.96 (0.71-1.29)	0.93 (0.69-1.24)	0.61 (0.49-0.75)	0.58 (0.50-0.68)	94 (93-96)	93 (92-94)	90 (88-92)	83 (81-85)
NSABP B-39/RTOG0413: Vicini et al,^12^ 2019; Tis-2 (≤3 cm); N0-1, M0									
WBI (50 Gy/25 fx)	2039	1 [Reference]	1 [Reference]	1 [Reference]	1 [Reference]	97 (96-97)	96 (95-97)	83 (81-84)	70 (68-72)
APBI	2093	1.1 (0.88-1.36)	1.05 (0.85-1.29)	0.72 (0.62-0.83)	0.76 (0.68-0.85)	96 (95-97)	96 (95-97)	88 (87-90)	77 (75-79)
**Localized prostate cancer**									
CHHiP: Dearnaley et al,^13^ 2016; T1b-3a, N0, M0; low, intermediate, and high risk									
RT (74 Gy/37 fx)	1065	1 [Reference]	1 [Reference]	1 [Reference]	1 [Reference]	93 (91-94)	92 (90-94)	77 (74-80)	65 (63-68)
RT (60 Gy/20 fx)	1074	0.73 (0.53-1.01)	0.72 (0.53-0.98)	0.75 (0.62-0.90)	0.87 (0.75-1.01)	95 (94-96)	95 (93-96)	83 (81-85)	70 (67-73)
HYPO-RT-PC: Widmark et al,^14^ 2019; T1c-3a, N0, M0; intermediate and high risk									
RT (78 Gy/39 fx)	591	1 [Reference]	1 [Reference]	1 [Reference]	1 [Reference]	96 (95-98)	96 (94-97)	80 (76-83)	68 (64-72)
RT (42.7 Gy/7 fx)	581	1.09 (0.72-1.66)	0.99 (0.66-1.49)	0.54 (0.41-0.71)	0.60 (0.48-0.75)	94 (92-96)	94 (92-96)	89 (86-91)	79 (76-83)

Abbreviations: APBI, accelerated partial breast irradiation; fx, fractions; HR, hazard ratio; CHHiP, Conventional or Hypofractionated High Dose Intensity Modulated Radiotherapy in Prostate Cancer; HYPO-RT-PC, Ultra-Hypofractionated vs Conventionally Fractionated Radiotherapy for Prostate Cancer; OCOG, Ontario Clinical Oncology Group; NSABP, National Surgical Adjuvant Breast and Bowel Project; RT, radiotherapy; Tam, tamoxifen alone; TamRT, tamoxifen plus radiotherapy; TME, total mesorectal excision; WBI, whole breast irradiation.

^a^ Baseline estimates are reconstructed from individual patient–level data without COVID-19 risk.

^b^ Infection risk per fraction of radiotherapy, 0.5%; case fatality rate, 5%.

^c^ Infection risk per fraction of radiotherapy, 5%; case fatality rate, 20%.

^d^ Infection risk per fraction of radiotherapy, 10%; case fatality rate, 30%.

To evaluate the association of different radiation fractionation schemes with OS in rectal cancer, we reanalyzed the TROG 01.04 trial,^8^ which randomized patients with T3N0-2M0 rectal cancer to short-course RT, consisting of 25 Gy in 5 fractions, vs long-course chemoradiation, consisting of 50.4 Gy in 28 fractions with concurrent 5-fluorouracil. We observed an association with improved survival in the short-course arm across a wide range of pandemic scenarios (eFigure 2 in the Supplement), including in scenarios with lower infection rates (IRF <5%) when CFR was in the 20%-30% range.

### Radiation Hypofractionation and Omission in Breast Cancer

**Figure 2.**
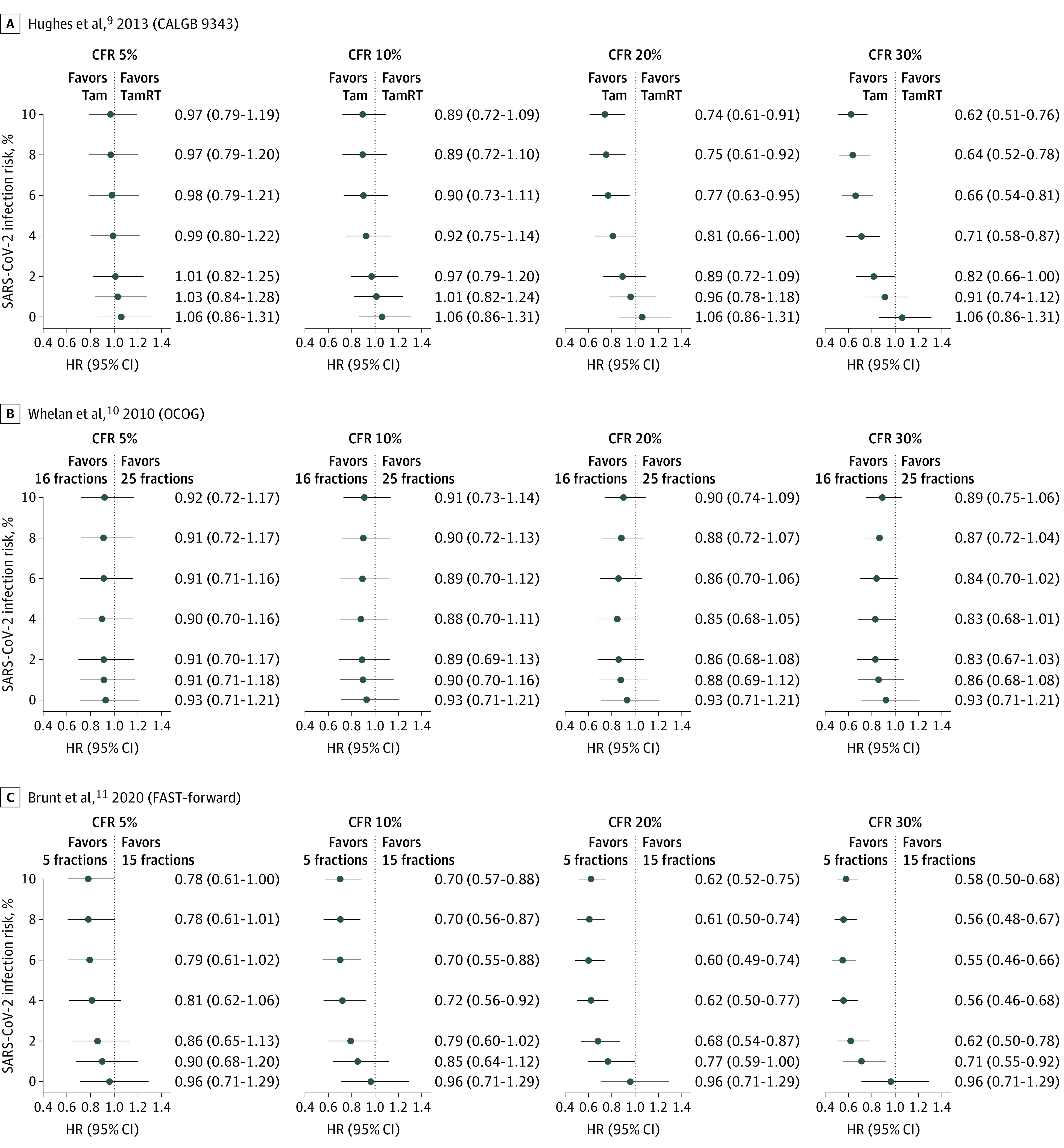
Estimated Median Hazard Ratios (HRs) Under a Range of Pandemic Scenarios Based on Simulations in Early Stage Breast Cancer HR estimates at 0% infection risk are from reconstructed data sets based on the original publications. All other HR estimates and 95% CIs are the median values from 25 000 simulations. CALGB indicates Cancer and Leukemia Group B; CFR, case fatality rate; OCOG, Ontario Clinical Oncology Group; Tam, tamoxifen; and TamRT, tamoxifen and radiation therapy.

The CALGB 9343 trial^9^ compared conventionally-fractionated whole breast RT and tamoxifen vs tamoxifen alone for women aged 70 years or older with T1N0, estrogen receptor–positive breast cancer, demonstrating that adjuvant RT can be reasonably omitted in this cohort. In our analysis with simulation of COVID-19 risk, adjuvant RT was associated with a survival detriment in most pandemic scenarios (Figure 2A; Table) with a median HR of 0.62 (median 95% CI, 0.51-0.76) in favor of tamoxifen alone in scenario C.

The OCOG hypofractionation trial^10^ and the UK START trials^26^ helped establish moderate hypofractionation as the standard of care for most patients receiving adjuvant RT to the breast alone. Reanalysis of the OCOG trial demonstrated no association between moderate hypofractionation and improved outcomes across all scenarios examined (Figure 2B; Table). COVID-19–associated risk increased to a similar extent in both the conventional and hypofractionated arms with higher pandemic risk, with median 5-year OS of 67% (95% CI, 64%-71%) in the conventional arm and 71% (95% CI, 67%-75%) in the hypofractionated arm , compared with 94% (95% CI, 92%-96%) in scenario C at baseline (Table).

The FAST-Forward trial^11^ investigated more aggressive hypofractionation in breast cancer, and demonstrated noninferiority of 26 Gy in 5 fractions adjuvant RT to the breast or chest wall vs standard hypofractionation to 40 Gy in 15 fractions. In contrast to the OCOG hypofractionation study, in simulations of the FAST-Forward trial,^11^ fewer fractions were associated with decreased COVID-19–associated mortality across many pandemic scenarios (Figure 2C; Table). However, in the highest-risk scenario (scenario C), COVID-19 mortality was sufficiently high to alter survival outcomes in both arms (median 5-year OS: 15 fractions, 72% [95% CI, 70%-74%]; and 5-fractions: 83% [95% CI, 81%-85%]; Table). This pattern was also reflected in the NSABP B-39 trial^12^ which compared APBI vs whole breast irradiation using conventional fractionation. APBI was associated with a benefit across most pandemic scenarios (Table; eFigure 3 in the Supplement) except for the highest risk scenarios.

### Radiation Hypofractionation in Prostate Cancer

**Figure 3.**
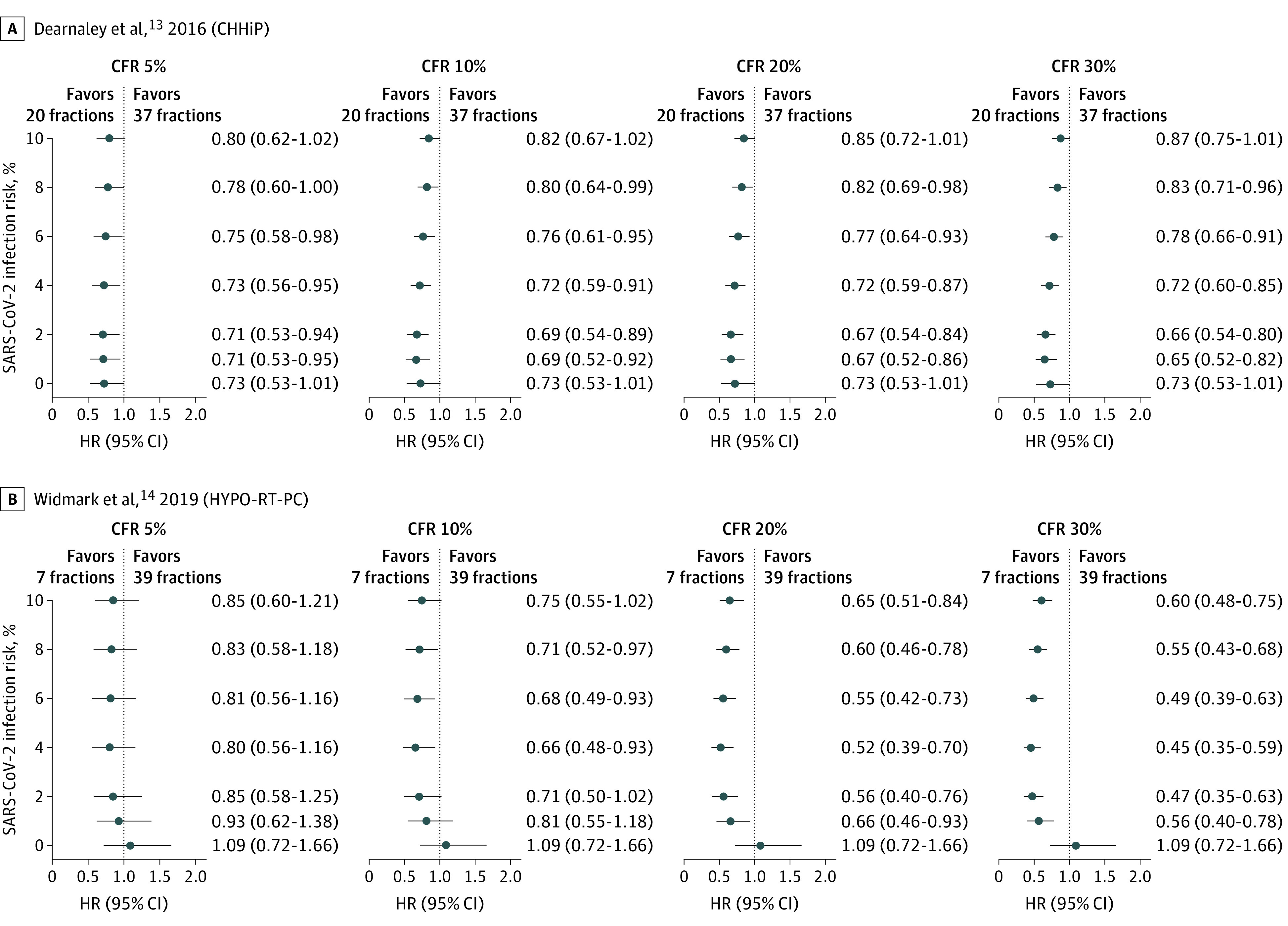
Estimated Hazard Ratios (HRs) Under a Range of Pandemic Scenarios Based on Simulations in Localized Prostate Cancer HR estimates at 0% infection risk are from reconstructed data sets based on the original publications. All other HR estimates and 95% CIs are the median values from 25 000 simulations. CFR indicates case fatality rate; CHHiP, Conventional or Hypofractionated High Dose Intensity Modulated Radiotherapy in Prostate Cancer; and HYPO-RT-PC, Ultra-Hypofractionated vs Conventionally Fractionated Radiotherapy for Prostate Cancer.

Moderate hypofractionation in prostate cancer has been tested in several recent trials (CHHiP,^13^ RTOG0415,^27^ HYPRO^28^). The CHHiP trial demonstrated noninferiority of 20 fractions vs 37 fractions in treatment of localized, primarily low- and intermediate-risk, prostate cancer.^13^ The trial included a 19-fraction arm that did not reach noninferiority, and we did not include it in our analysis. In simulations with varying COVID-19 risk, the 20-fraction regimen was not associated with changes in HR for OS (Figure 3A, Table).

HYPO-RT-PC^14^ was a noninferiority trial of 78 Gy in 39 fractions vs 42.7 Gy in 7 fractions in intermediate- and high-risk prostate cancer. On reanalysis, the estimated HR changed from 1.09 (95% CI, 0.72-1.66) at baseline to a median HR of 0.99 (median 95% CI, 0.66-1.49) in the lowest-risk scenario (scenario A) in favor of the hypofractionated arm (Figure 3B, Table). In higher risk scenarios, the hazard ratio improved even further, and in most scenarios the median 95% CI excluded 1.0 in favor of the hypofractionated arm (Figure 3B).

### Risk Associated With Number of Fractions

**Figure 4.**
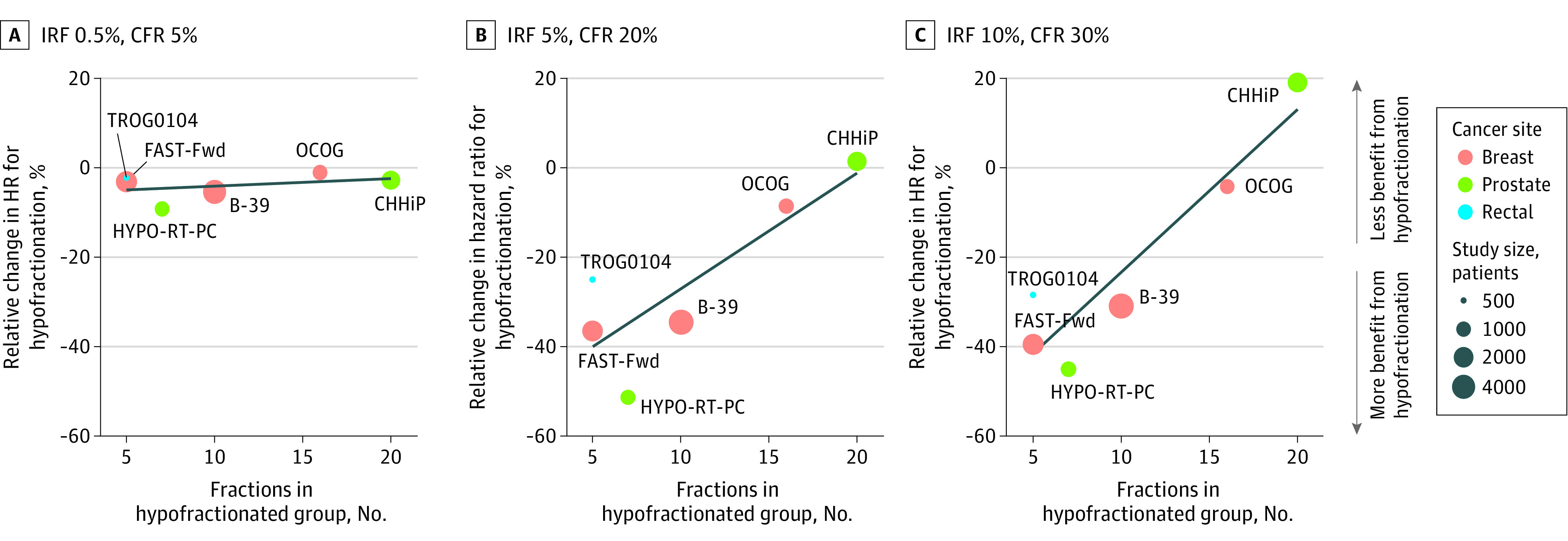
Association of Number of Fractions in Hypofractionated Arm and Magnitude of Change in Hazard Ratio From Baseline Under Different Pandemic Scenarios for Hypofractionation Trials in Breast Cancer, Prostate Cancer, and Rectal Cancer B-39 indicates National Surgical Adjuvant Breast and Bowel Project B-30; CFR, case fatality rate; IRF, infection risk per fraction; CHHiP, Conventional or Hypofractionated High Dose Intensity Modulated Radiotherapy in Prostate Cancer; FAST-Fwd, FAST-Forward; HR, hazard ratio; HYPO-RT-PC, Ultra-Hypofractionated vs Conventionally Fractionated Radiotherapy for Prostate Cancer; and OCOG, Ontario Clinical Oncology Group.

Figure 4 shows the association between the number of fractions in the hypofractionated regimen and magnitude of change in estimated HR from the baseline estimate across 3 pandemic scenarios. In lower-risk scenarios (scenario A), the degree of hypofractionation was not associated with the HR for OS compared with the baseline. However, with higher COVID-19–associated risks (scenarios B and C), trials with a greater degree of hypofractionation (ie, 5-10 fractions) were associated with a larger magnitude of improvement in survival outcomes in favor of the hypofractionated arm.

## Discussion

In this comparative effectiveness study, we developed an estimation procedure for incorporating COVID-19 risk associated with RT, and reanalyzed RCTs in breast, prostate, and rectal cancers with different fractionation regimens to assess COVID-19–associated risk. We describe the expected association of COVID-19 with OS based on daily risk of being infected by SARS-CoV-2 during RT (IRF) and the resulting risk of death from infection (CFR). While we specifically examined 3 possible risk scenarios for illustration, the model can be extended to varying scenarios. To our knowledge, our study is the first to attempt to quantify these risks in a model incorporating RCT data and varying COVID-19 risk. With such a design, we are able to appreciate the differential benefits of radiation hypofractionation in different settings.

The COVID-19 pandemic has created unprecedented challenges in delivery of cancer therapy. Oncologists face a difficult risk-benefit calculus in weighing risk of infection against risk of cancer progression. To help provide guidance to clinicians, several groups have released guidelines on cancer therapy in the COVID-19 era.^4^ While expert opinions and consensus guidelines are valuable, real-time clinical decisions can also be aided by quantitative tools that can provide estimates of outcomes based on varying risk, tailored to a specific setting and patient. Such quantitative models can facilitate an adaptive approach to weigh risks and benefits of treatment regimens relative to the dynamic risk of COVID-19, and serve an important complementary role to expert-derived guidelines.

In a setting with lower risks, there were minimal changes to treatment effect estimates. As risk mitigation strategies have been implemented,^29^ the lower-risk scenario may be applicable to most clinical settings. Thus, our results support the continuation of necessary clinical care for patients receiving cancer care in these settings, despite a general trend during COVID-19 toward deescalating or postponing care and a sharp decline in cancer screening.^30^ These findings are supported by an initial study by Xie et al^31^ on RT delivery during the COVID-19 pandemic that reported a very low infection rate of 0.5% but in which more than 50% of patients experienced disruption of RT.

In higher risk settings, the use of hypofractionated RT courses was associated with decreased COVID-19 mortality risk, and the degree of hypofractionation was associated with magnitude of benefit. Breast and prostate cancer trials provide illustrative examples. Even in higher-risk pandemic scenarios, moderate hypofractionation in breast cancer was not associated with improvement in survival, likely since the small relative reduction in number of fractions was not sufficient to substantially affect survival. Regardless, moderate hypofractionation represents the appropriate standard of care vs conventional fractionation for eligible patients.^32^ In higher-risk scenarios, more abbreviated courses with 5-fraction RT or APBI were associated with better survival outcomes. Furthermore, in scenarios in which treatment may be omitted, such as in patients older than 70 years with T1N0 estrogen receptor–positive breast cancer, our results support omission of treatment and the associated exposure risk.

We observed a similar pattern in localized prostate cancer, in which moderate hypofractionation was not associated with a survival advantage in the CHHiP trial, but more aggressive hypofractionation in the HYPO-RT-PC trial was associated with a benefit in higher-risk scenarios. While shorter regimens should be considered, concerns remain about the potential long-term toxic effects of ultra-hypofractionated regimens. Delay of treatment with use of androgen deprivation therapy is another consideration and is supported by RCT data in some settings.^33,34^ While delaying RT may be reasonable, clinicians must weigh the uncertainty of evolving COVID-19 risks, as short-term delays may not be beneficial if local COVID-19 risks persist.

In rectal cancer, some published expert guidelines have recommended omission of preoperative RT in patients with cT3a/b and cN1-2 disease in the setting of COVID-19.^35^ In our reanalysis of the Dutch TME trial, the addition of short-course preoperative RT was not associated with a substantial change in survival in all but the highest-risk scenarios. Our findings thus support continued use of RT in most pandemic settings, given the known benefits with 50% reduction in local recurrence and an overall survival benefit for patients with stage III disease on subgroup analysis.^7^

### Limitations

Our study has several limitations. We did not model infection risks associated with other treatment modalities, such as surgery, chemotherapy, or immunotherapy. Our model could be extended to analyze these modalities by similarly incorporating infection and mortality risk associated with surgical or chemotherapy encounters into published RCTs. Further data on the association of specific therapies with COVID-19–related risks would allow for better modeling of these risks.^36,37^ In addition, infection risk from community spread, independent of health care system contact, would be a competing risk for survival outcomes. We did not examine the strategy of delaying patients, which is difficult to study given the paucity of RCTs evaluating treatment delay. We focused on survival outcomes, but there are other clinical end points, such as quality of life and local control, that are also relevant. Our analyses were based on reconstructed data, which are thought to be representative of published data but do not allow for incorporation of other covariates and risk factors for COVID-19, such as comorbidities, race/ethnicity, and body mass index.

## Conclusions

In this comparative effectiveness study, we developed a quantitative approach for assessing COVID-19–associated risk using RCTs. Our results suggest minimal mortality risk with standard treatment regimens when infection control measures can keep daily infection risk at or below 0.5% and support the delivery of standard evidence-based cancer care in most settings when appropriate precautions are taken. We demonstrate that the benefits of treatment modification vary based on the local pandemic scenario and degree of treatment shortening. Our model provides a framework to help guide clinical decision-making for cancer patients during the COVID-19 pandemic.

## Notes

Supplement. **eFigure 1.** Reconstructed Kaplan Meier Figures for Overall Survival **eFigure 2.** Estimated Median Hazard Ratios Under a Range of Pandemic Scenarios Based on Simulations of the Dutch TME and TROG01.04 Trials **eFigure 3.** Estimated Median Hazard Ratios Under a Range of Pandemic Scenarios Based on Simulations of the NSABP B-39 Trial
