# Supplementary material for: Assessment of Simulated SARS-CoV-2 Infection and Mortality Risk Associated With Radiation Therapy Among Patients in 8 Randomized Clinical Trials
Source: JAMA Netw Open. 2021 Mar 29;4(3):e213304. doi: 10.1001/jamanetworkopen.2021.3304 (PMC8008289; doi:10.1001/jamanetworkopen.2021.3304)
Supplement: Supplement. — eFigure 1. Reconstructed Kaplan Meier Figures for Overall Survival eFigure 2. Estimated Median Hazard Ratios Under a Range of Pandemic Scenarios Based on Simulations of the Dutch TME and TROG01.04 Trials eFigure 3. Estimated Median Hazard Ratios Under a Range of Pandemic Scenarios Based on Simulations of the NSABP B-39 Trial [file jamanetwopen-e213304-s001.pdf]

Supplemental Online Content

Tabrizi S, Trippa L, Cagney D, et al. Assessment of Simulated SARS-CoV-2 Infection and Mortality Risk Associated With Radiation Therapy Among Patients in 8 Randomized Clinical Trials. *JAMA Netw Open*. 2021;4(3):e213304. doi:10.1001/jamanetworkopen.2021.3304

eFigure 1. Reconstructed Kaplan Meier Figures for Overall Survival
eFigure 2. Estimated Median Hazard Ratios Under a Range of Pandemic Scenarios Based on Simulations of the Dutch TME and TROG01.04 Trials
eFigure 3. Estimated Median Hazard Ratios Under a Range of Pandemic Scenarios Based on Simulations of the NSABP B-39 Trial

This supplemental material has been provided by the authors to give readers additional information about their work.

© 2021 Tabrizi S et al. *JAMA Network Open.*

eFigure 1. Reconstructed Kaplan Meier Figures for Overall Survival

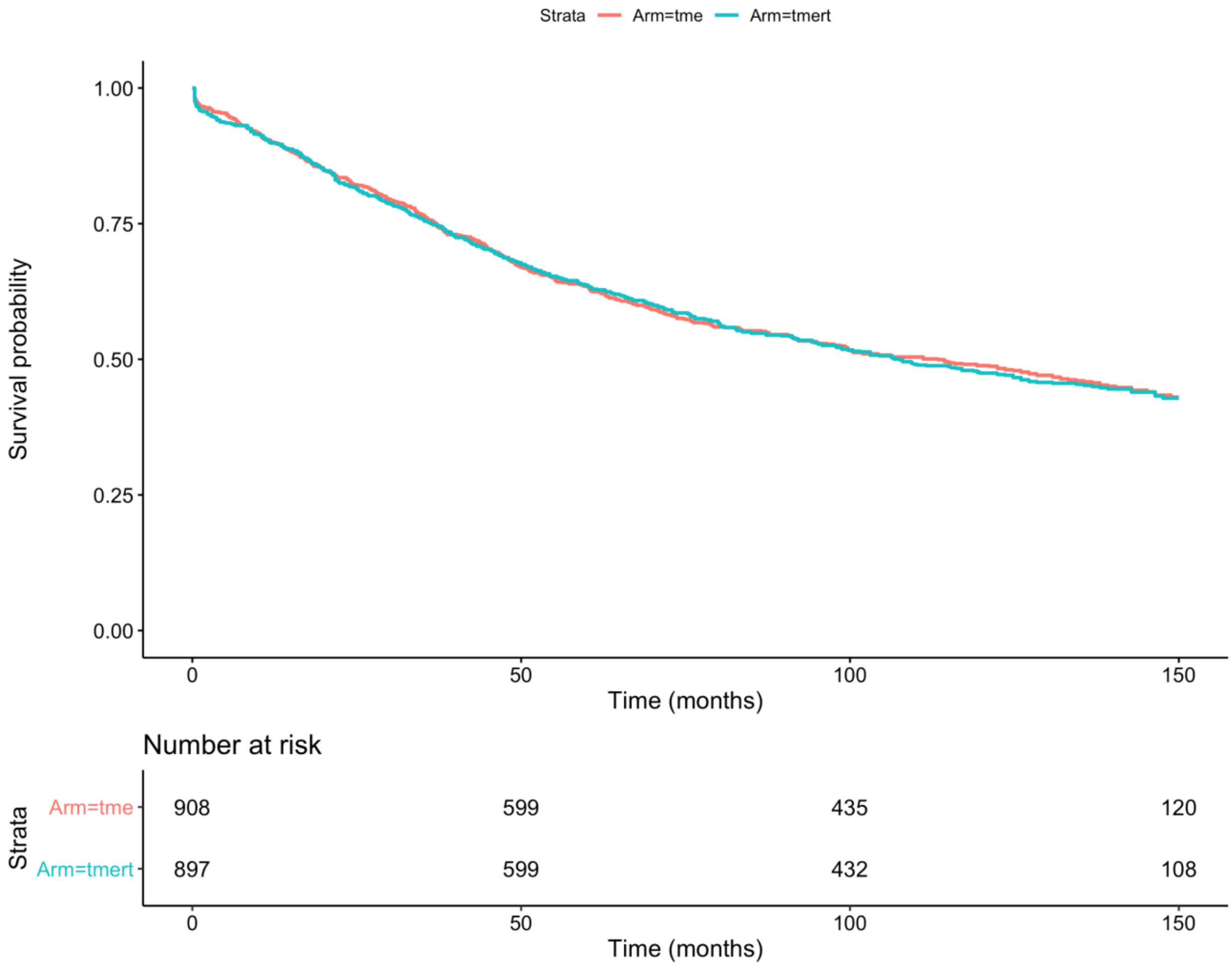

Reconstructed Kaplan-Meier figure for OS from the Dutch TME trial

© 2021 Tabrizi S et al. *JAMA Network Open.*

eFigure 1B

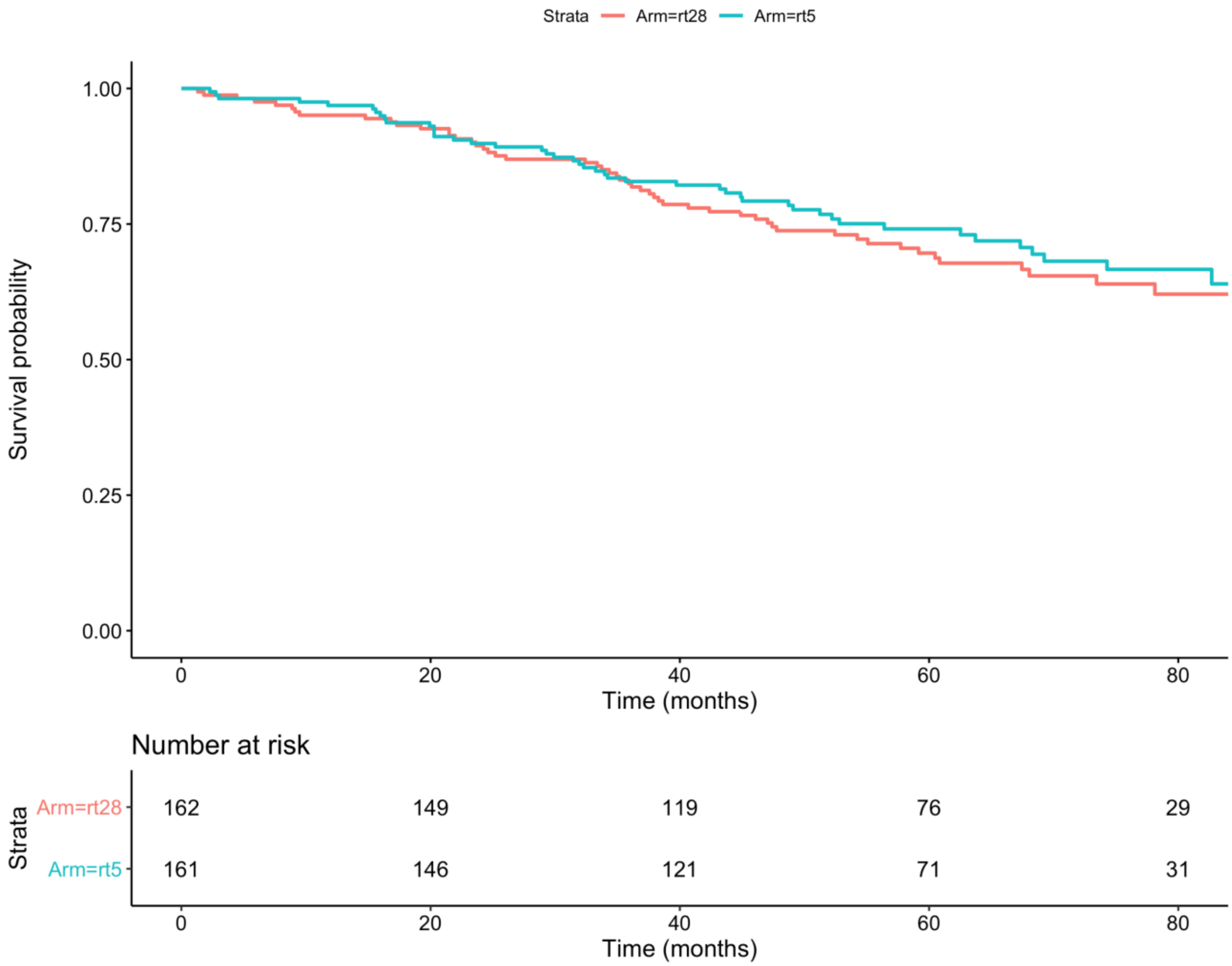

Reconstructed Kaplan-Meier figure for OS from TROG 01.04

© 2021 Tabrizi S et al. *JAMA Network Open.*

eFigure 1C

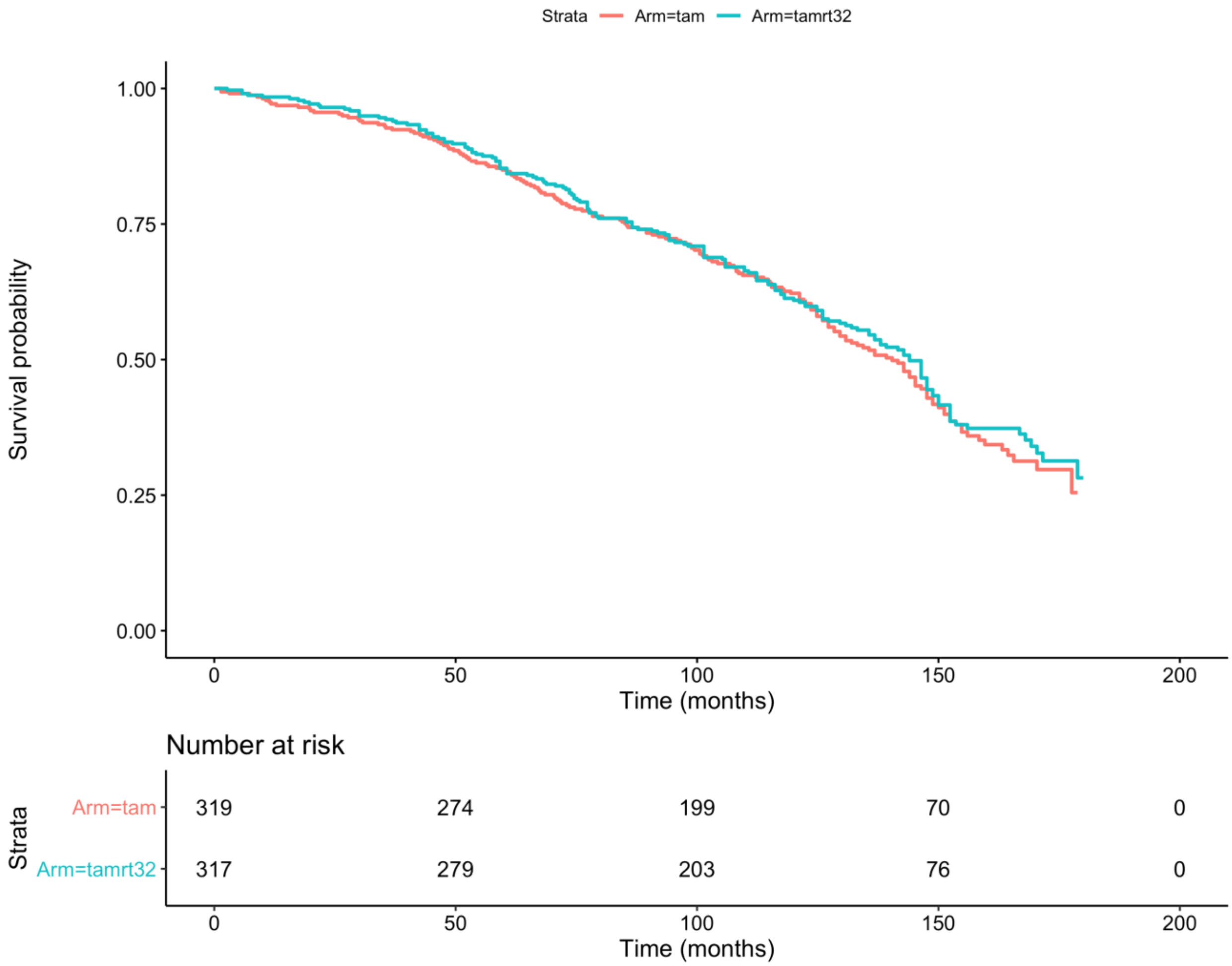

Reconstructed Kaplan-Meier figure for OS from CALGB 9343

© 2021 Tabrizi S et al. *JAMA Network Open.*

eFigure 1D

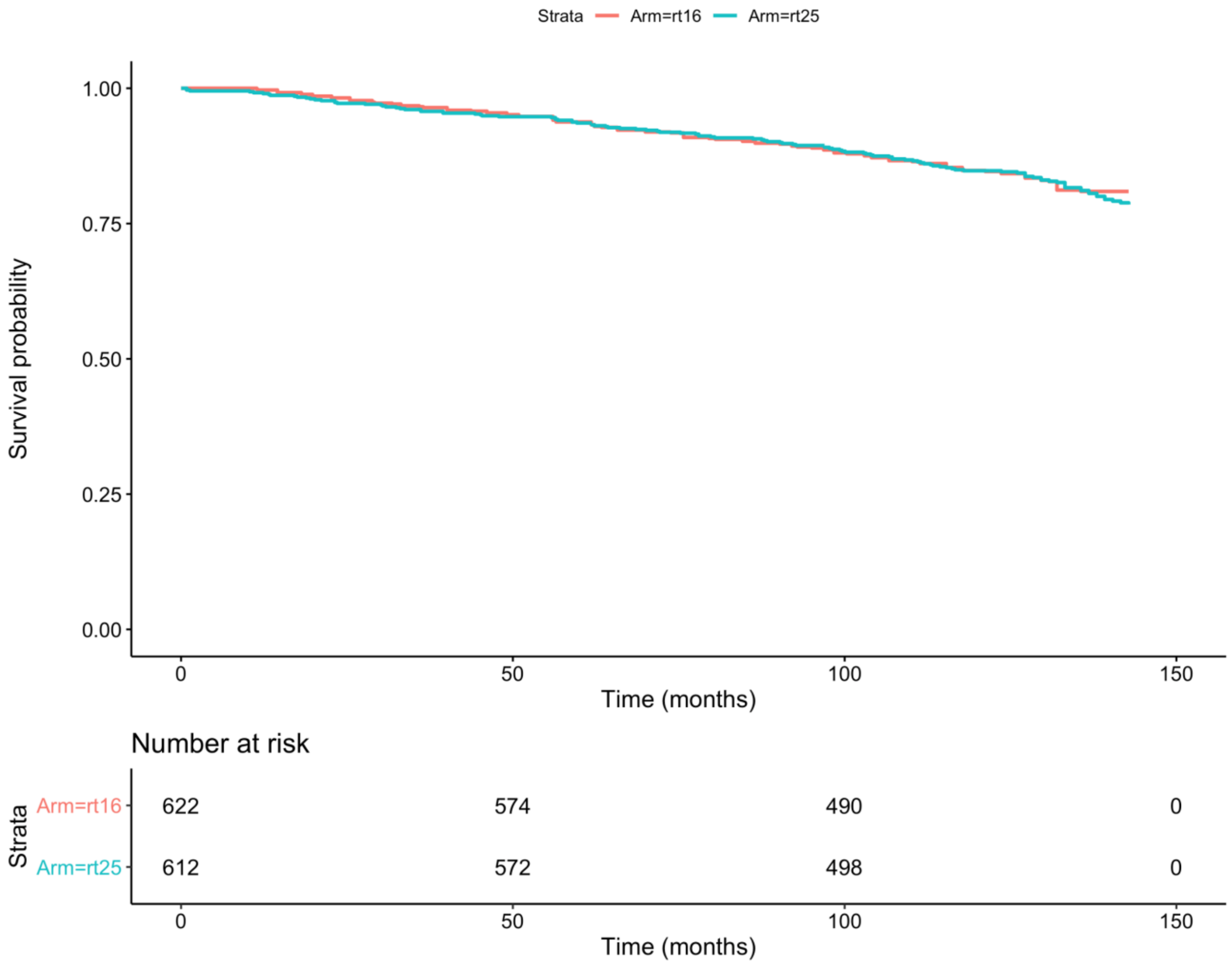

Reconstructed Kaplan-Meier figure for OS from OCOG hypofractionation trial in breast cancer

© 2021 Tabrizi S et al. *JAMA Network Open.*

eFigure 1E

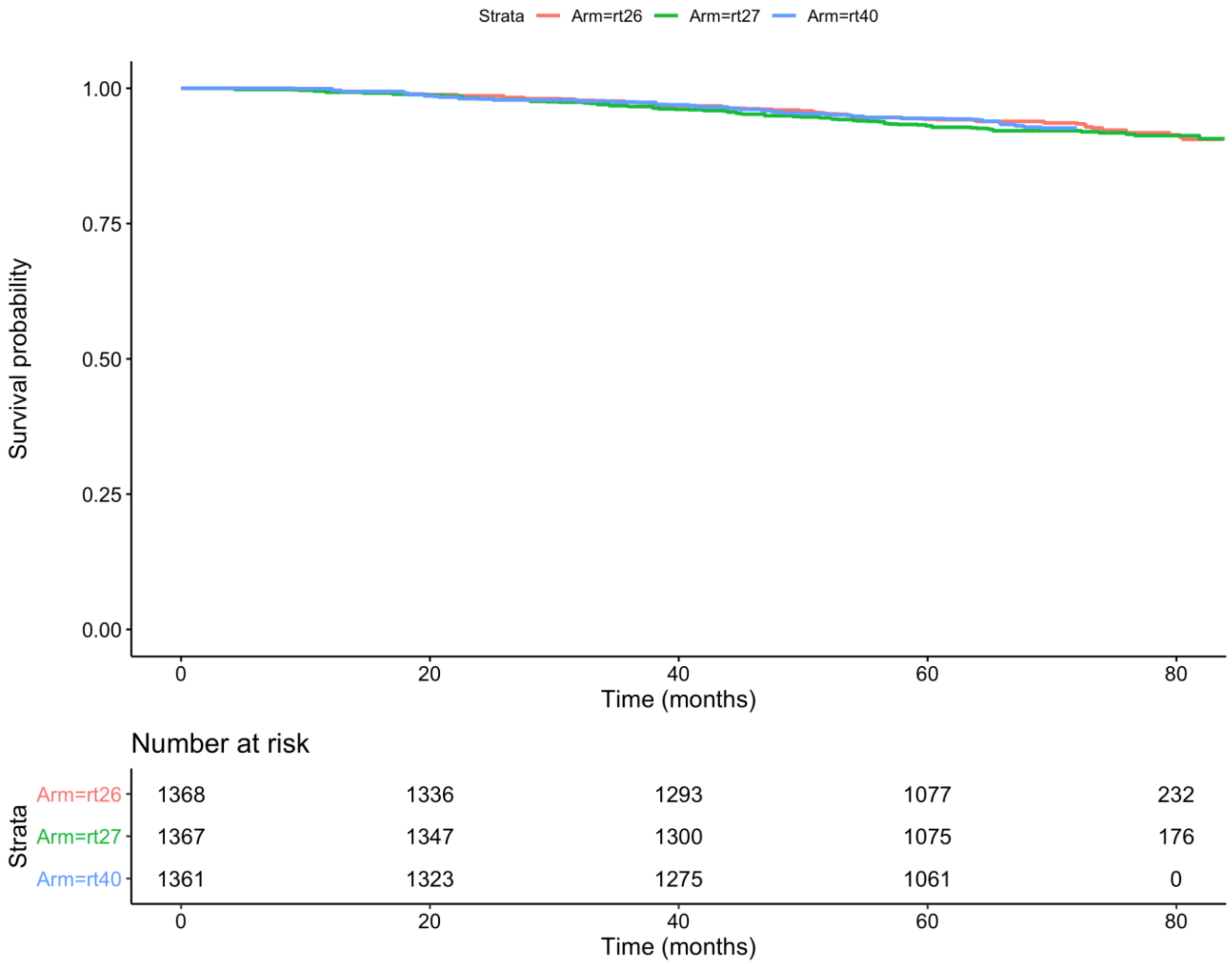

Reconstructed Kaplan-Meier figure for OS from FAST-Forward

© 2021 Tabrizi S et al. *JAMA Network Open.*

eFigure 1F

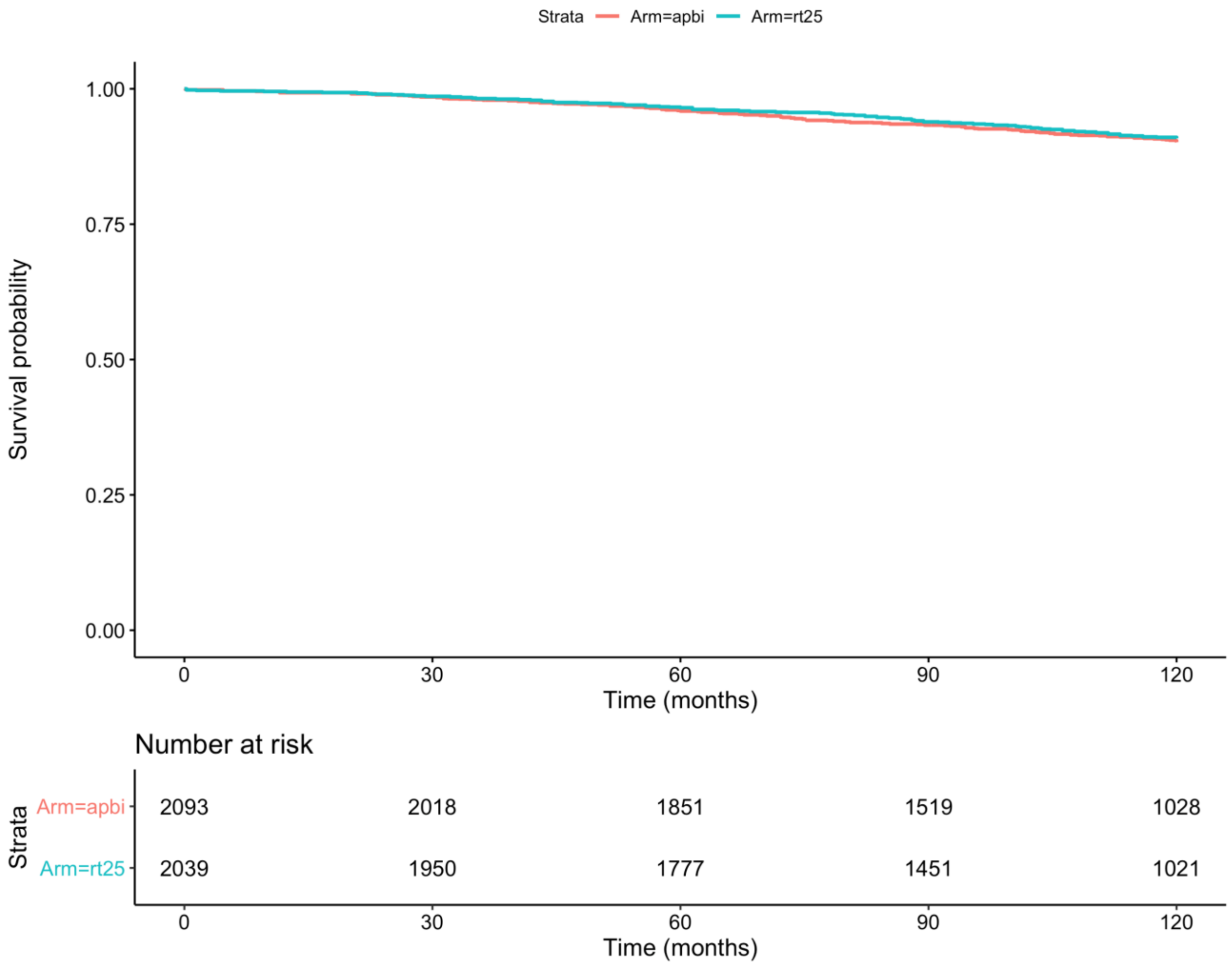

Reconstructed Kaplan-Meier figure for OS from RTOG0413/NSABP B39

© 2021 Tabrizi S et al. *JAMA Network Open.*

eFigure 1G

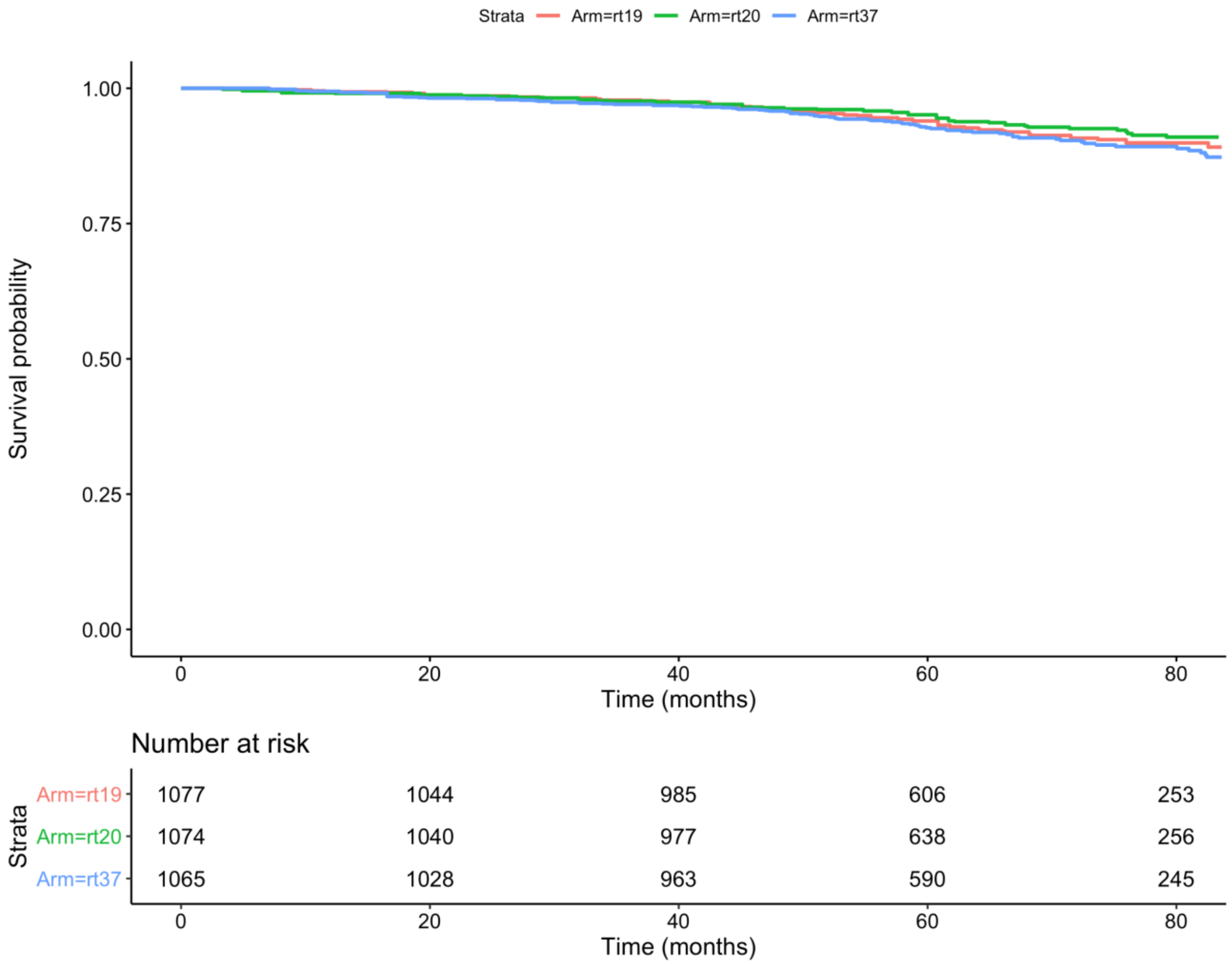

Reconstructed Kaplan-Meier figure for OS from CHHiP

© 2021 Tabrizi S et al. *JAMA Network Open.*

eFigure 1H

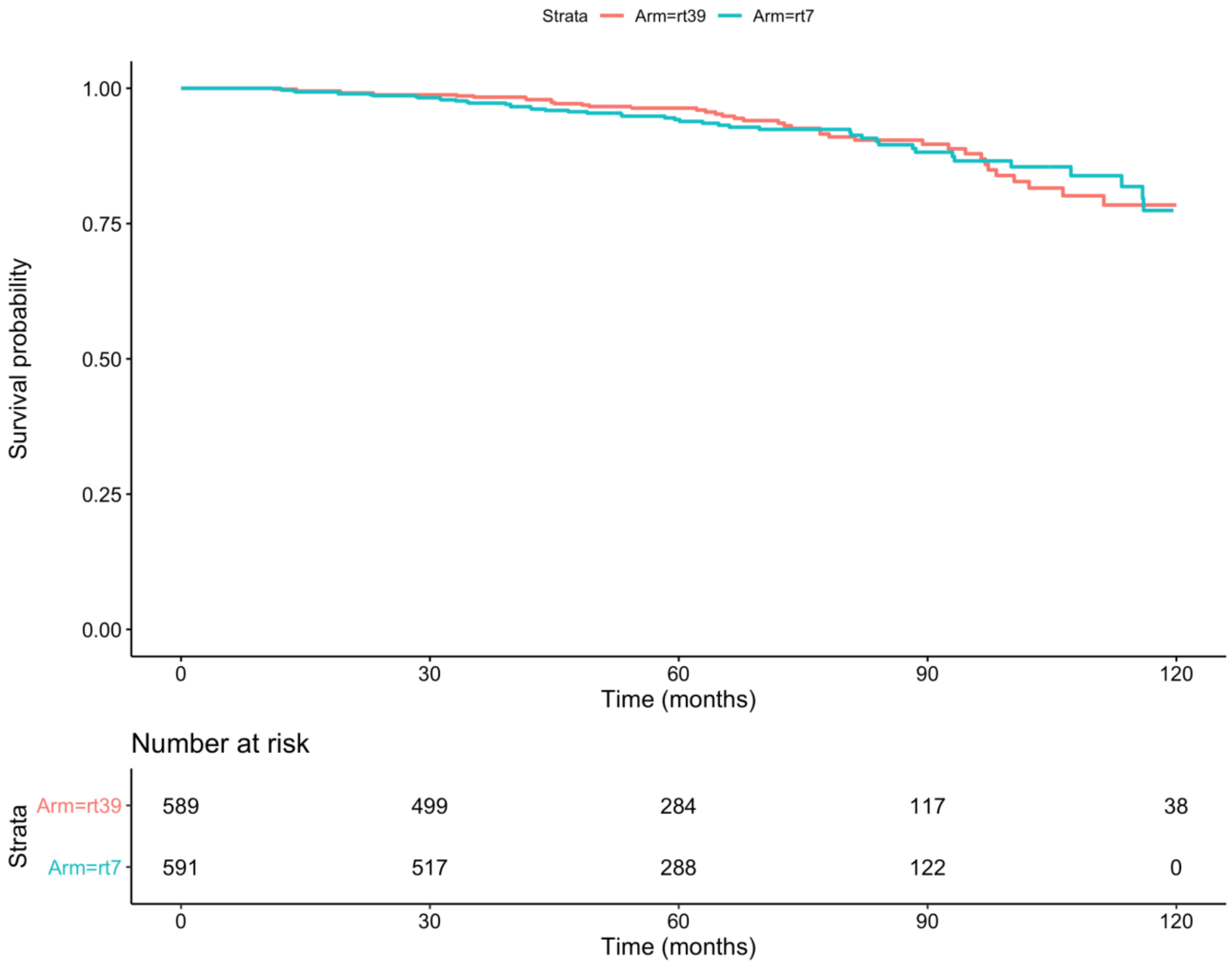

Reconstructed Kaplan-Meier figure for OS from HYPO-RT-PC

© 2021 Tabrizi S et al. *JAMA Network Open.*

eFigure 2. Estimated Median Hazard Ratios Under a Range of Pandemic Scenarios Based on Simulations of the Dutch TME and TROG01.04 Trials

A

Van Gijn et al (Dutch TME)[7]

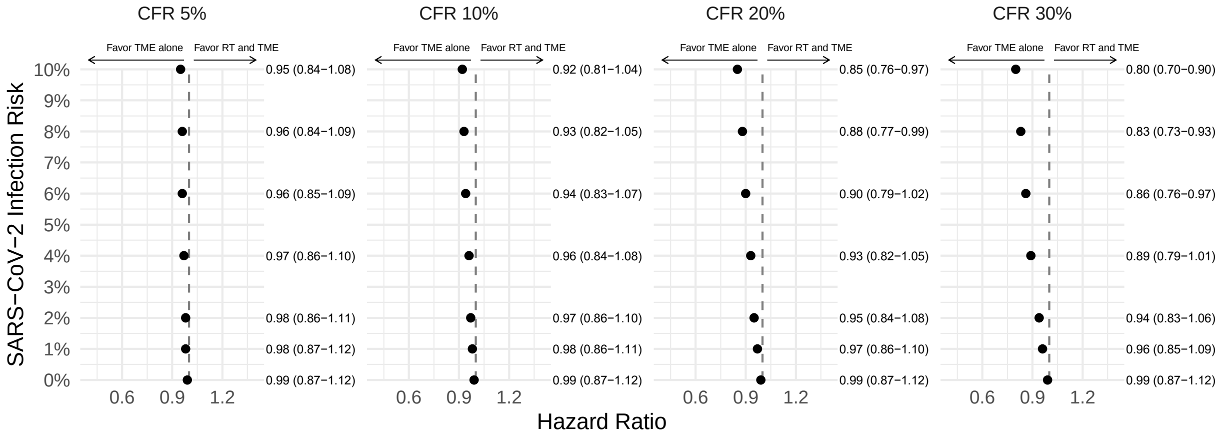

B

Ngan et al (TROG01.04)[8]

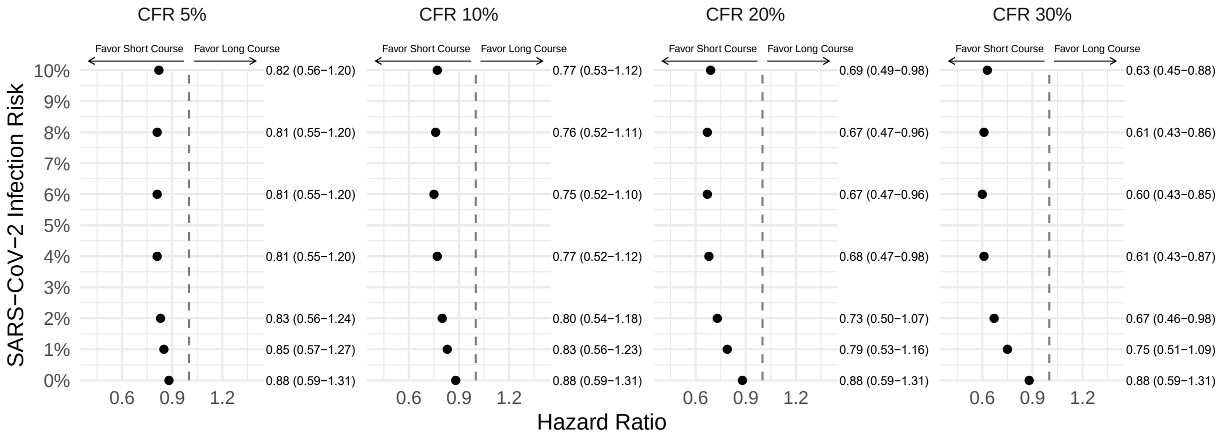

Estimated median hazard ratios and 95% confidence-intervals under a range of pandemic scenarios based on simulations of the Dutch TME (A) and TROG01.04 (B) trials. Hazard Ratio (HR) estimates at 0% infection risk are from reconstructed datasets based on the original publications. All other HR estimates and 95% confidence-intervals are the median values from 25,000 simulations. RT: Radiation Therapy. TME: Total Mesorectal Excision. CFR: Case Fatality Rate.

© 2021 Tabrizi S et al. *JAMA Network Open.*

eFigure 3. Estimated Median Hazard Ratios Under a Range of Pandemic Scenarios Based on Simulations of the NSABP B-39 Trial

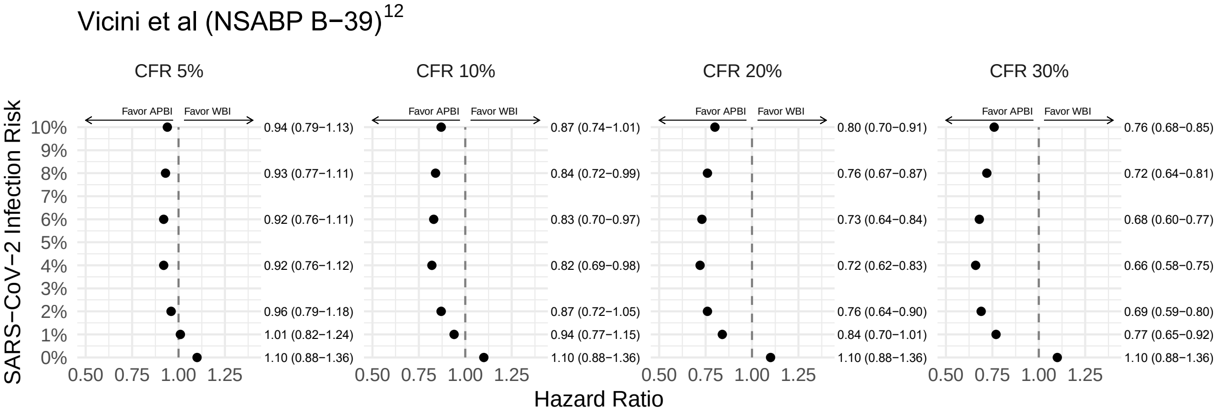

Estimated median hazard ratios and 95% confidence-intervals under a range of pandemic scenarios based on simulations of the NSABP B-39 trial. Hazard Ratio (HR) estimates at 0% infection risk are from reconstructed datasets based on the original publications. All other HR estimates and 95% confidence-intervals are the median values from 25,000 simulations. APBI: Accelerated Partial Breast Irradiation. WBI: Whole Breast Irradiation. CFR: Case Fatality Rate.

© 2021 Tabrizi S et al. *JAMA Network Open.*
